# Identification of Chemokines and Growth Factors in Proliferative Diabetic Retinopathy Vitreous

**DOI:** 10.1155/2014/486386

**Published:** 2014-10-27

**Authors:** Ying Dai, Zhifeng Wu, Feng Wang, Zhengwei Zhang, Mengxi Yu

**Affiliations:** Department of Ophthalmology, Wuxi No. 2 Hospital, Nanjing Medical University, Zhongshan 68, Wuxi 214002, China

## Abstract

Associations were investigated between levels of chemokines and growth factors in the vitreous and proliferative diabetic retinopathy (PDR). Enrolled were 58 patients (58 eyes) requiring pars plana vitrectomy (PPV), with PDR (*n* = 32, none with traction retinal detachment) or not (non-PDR). In the latter, 16 had macular hole (MH) and 10 had epiretinal membrane (ERM). With a multiplex bead immunoassay, levels of 11 chemokines and growth factors were measured from the undiluted vitreous sample from each patient. In the non-PDR eyes, the levels of the 11 chemokines and growth factors tested were similar between patients with MH and those with ERM. However, the levels of all 11 were significantly higher in the PDR eyes relative to the non-PDR; CCL17, CCL19, and TGF*β*3 were markedly upregulated and have not been investigated in PDR previously. The significantly higher levels of CCL4 and CCL11 in PDR contradict the results of previous reports. Based on Spearman's nonparametric test, moderate-to-strong correlations were found between VEGF and other mediators. Our results indicate that these chemokines and growth factors could be candidates for research into targeted therapies applied either singly or in combination with anti-VEGF drugs for the treatment of PDR.

## 1. Introduction

Proliferative diabetic retinopathy (PDR) is the most serious complication of diabetic microvascular disorders. Of note, PDR is characterized by retinal neovascularization and fibrovascular proliferation [1], which should be responsible for the occurrence of vitreous hemorrhage (VH) and traction retinal detachment (TRD) [2–4]. It is therefore reasonable to suppose that, in a worst-case scenario, PDR could contribute to irreversible vision loss or even blindness [4].

Currently, retinal photocoagulation is considered an effective treatment for PDR because of its role in the regression of present neovascularization, prevention of neovascularization regeneration, and reduction of macular edema [5]. Another treatment option is pars plana vitrectomy (PPV) with removal of vitreous hemorrhage and fibrovascular tissue [5]. Furthermore, an increasing number of investigators have been concerned with the development of antivascular endothelial growth factor (VEGF) drugs for the treatment of retinal neovascular diseases [6–8]. Additionally, several studies have found that short-term benefits could be obtained in the majority of PDR patients through anti-VEGF treatment [2, 9, 10]. However, anti-VEGF agents in the vitreous could pass into the circulation and subsequently result in adverse systemic effects including hypertension and proteinuria [11–13]. Also, ocular adverse effects, such as endophthalmitis and retinal detachment, could occur after administration by intravitreal injection [2, 11, 12, 14]. Herein, novel therapeutic strategies are required to avoid the adverse side effects of anti-VEGF agents.

To date, VEGF is widely recognized as a considerably effective candidate for angiogenesis formation [11]. A great number of mediators as well as VEGF were observed at raised levels in the vitreous of patients with retinal neovascular diseases, such as PDR [2, 15]. Besides recruitment of leukocytes and promotion of local inflammation, chemokines, known as multifunctional molecules, could participate in the regulation of angiogenesis [1, 16]. Moreover, the involvement of growth factors, such as TGF*β*2, has been demonstrated in association with retinal neovascularization [3]. Thus, we assume that abnormally elevated chemokines and growth factors would be involved in the modulation of retinal neovascularization of PDR patients. Such chemokines and growth factors may be viable targets of potential therapies, therapies that could be administered solely or in combination with anti-VEGF agents.

In an attempt to verify our hypothesis, we measured 11 different kinds of chemokines and growth factors in undiluted vitreous obtained from patients with PDR. Through multiplex bead analysis, we screened out three novel candidate mediators that were reportedly involved in PDR. In addition, we identified two other significantly increased mediators, whose results have been previously controversial. These analytes may be therapeutic targets in PDR, particularly for patients exhibiting side effects to anti-VEGF treatment.

## 2. Materials and Method

### 2.1. Study Population

The local ethics committee approved this study, which was conducted in accordance with the tenets of the Helsinki Declaration. All participants read carefully and signed an informed consent form prior to PPV.

Fifty-eight patients (58 eyes) requiring PPV were enrolled in this study. Among them, 32 eyes of 32 individuals received diagnoses of PDR. Twenty-six eyes of 26 patients were without retinal neovascular diseases and served as controls (non-PDR group), and, of these, 16 patients had macular hole (MH) and 10 had epiretinal membrane (ERM).

Upon admission, all participates underwent a detailed fundus examination and medical histories were recorded. Moreover, specialized examinations were performed by at least two experienced ophthalmologists and all surgeries were carried out by the same person of our department. Of the 32 PDR patients, the most had suffered from diabetes mellitus for over one decade. Approximately 91% of the PDR patients had received long-term regular insulin treatment under the guidance of endocrinologists. Of note, about seven eighths of PDR patients had received retinal photocoagulation one or more times previously. None of the PDR patients was complicated with TRD and none of the controls suffered from diabetes or retinal detachment. In addition, none of the participants had ever been treated with anti-VEGF agents prior to the PPV.

Patients' demographics were collected and illustrated in Table 1. The clinical variables recorded were age, gender, duration of diabetes mellitus, fasting blood-glucose, glycosylated hemoglobin, and medical history.

### 2.2. Sample Collection

During the PPV and before fluid infusion, the undiluted vitreous samples were aspirated with 5 mL syringes during PPV. Approximately 1.2–1.5 mL vitreous was obtained per eye. The vitreous samples were placed in 1.5 mL polypropylene tubes on ice immediately. After centrifugation at 3000 rpm for 10 minutes at 4°C, the supernatants were stored at −80°C until analyzed.

### 2.3. Multiplex Bead Immunoassay

A multiplex bead immunoassay (EMD Milliplex, Milliplex, Billerica, MA, USA) was performed to analyze levels of a wide range of 11 chemokines and growth factors in undiluted vitreous obtained from PDR, MH, and ERM patients using Luminex 200 instrumentation (Luminex Corporation, Austin, TX, USA). Compared to traditional enzyme linked immunosorbent assay (ELISA), multiplex bead immunoassay is characterized by higher sensitivity, a wider detection range, and better accuracy and repeatability. Surprisingly, this new technique allowed for the measurement of 96 samples simultaneously and the detection of up to 100 profiles per sample.

In brief, standards, controls, vitreous samples, assay buffer, matrix solution, and antibody-coated microspheres were added to the appropriate wells. After incubation overnight at 4°C with shaking, the 96-well plates were washed twice with wash buffer. Detection antibodies were added per well and incubated for an hour. When the incubation with streptavidin-phycoerythrin was completed, the 96-well plates were washed again and read on Luminex. Every assay was performed in accordance with the manufacturer's instructions. Of note, to measure TGF*β* isoforms, before the experiment, all samples were prepared for the acidizing treatment. Chemokines and growth factors levels over the maximum detection limit were recorded as the maximum value and vice versa. The 11 mediators and their detection ranges were listed in Table 2.

### 2.4. Statistical Analysis

Nonnormally distributed data, such as levels of mediators, were analyzed using the Mann-Whitney *U* test. Also employed was the chi-square test for clinical variables such as gender. Correlations between VEGF and the other profiles were explored with Spearman's nonparametric test. All data were analyzed using SPSS 17.0 software (Chicago, IL, USA). The photographs were created with Graphpad Prism 5.0 (La Jolla, CA, USA). A *P* value < 0.05 was considered statistically significant.

## 3. Result

### 3.1. Clinical Result

Clinical variables were available for all enrolled participants separated into PDR and non-PDR group. The PDR cases were composed of 18 males (56%) and 14 females (44%) with an average age of 58 years. In non-PDR group, there were 12 males (46%) and 14 females (54%) with an average age of 58 years. The differences in gender (*P* = 0.598) and age (*P* = 0.851) were not significant between the groups. In the non-PDR group of patients with MH (*n* = 16), the genders were equally divided. Of the 10 patients in the non-PDR with ERM, 6 were women. None of the non-PDR participants had a history of diabetes mellitus and none had received insulin or laser treatment. Regarding fasting blood-glucose and glycosylated hemoglobin, significant difference was found between PDR and non-PDR groups (*P* < 0.001, *P* < 0.001, resp.).

### 3.2. Chemokines Levels and in Vitreous

Through univariate logistic regression, no obviously statistical differences in mediators' levels were found between the patients with MH and those with ERM. Thus, it appeared to be appropriate to classify MH together with ERM patients as non-PDR cases. The chemokines levels were concluded in Table 3(a).

**Figure 1:**
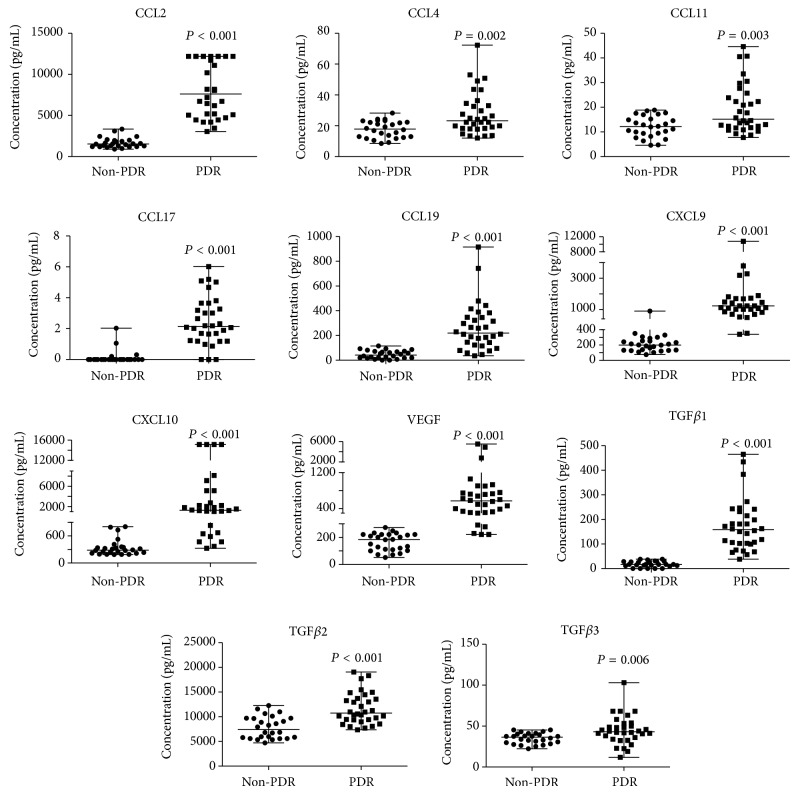
Scatter plots of chemokines and growth factors levels in PDR vitreous. By multiplex beads immunoassay, significant elevation of all 11 mediators was found in PDR vitreous as compared with controls. Sixteen MH and 10 ERM patients constituted the non-PDR group. Among 11 mediators, CCL17, CCL19, and TGF*β*3 were identified to be associated with PDR firstly. The vertical bars represent median with range.

Expect for CCL17, all the mediators tested could be detected in the vitreous of MH, ERM, and PDR patients. In the PDR patients, the CCL2 levels exceeded the maximum detection limit in approximately 31% (10/32) of samples. According to the results from the nonparametric Mann-Whitney test, all of the chemokines tested were found at significantly higher levels in the PDR group than in the non-PDR. CCL17 and CCL19 have never been investigated in PDR vitreous before, and we found that while CCL17 was present at low levels in PDR patients; this chemokine was undetectable in the majority of the non-PDR patients. Regarding CCL19, an obvious difference was found in the vitreous with median levels of 219.67 pg/mL in PDR patients but 41 pg/mL in controls. Associations between CCL4 and PDR and CCL11 and PDR have previously been equivocal [2, 15, 17]. In the present study, the median levels of CCL4 and CCL11 in the PDR group were approximately 1.3-fold and 1.2-fold, respectively, that of the controls (Figure 1).

### 3.3. Growth Factors Levels in Vitreous

The growth factors levels were summarized in Table 3(b). In all the vitreous samples analyzed, detectable levels of all growth factors were found. Significant differences were observed in the VEGF and TGF-*β* isoforms. Among the growth factors tested, only TGF-*β*3 was the first to be investigated for an association with PDR. We found that the median TGF-*β*3 levels in the PDR patients were 1.1-fold that of the non-PDR patients (Figure 1).

### 3.4. Correlations between Mediators

**Figure 2:**
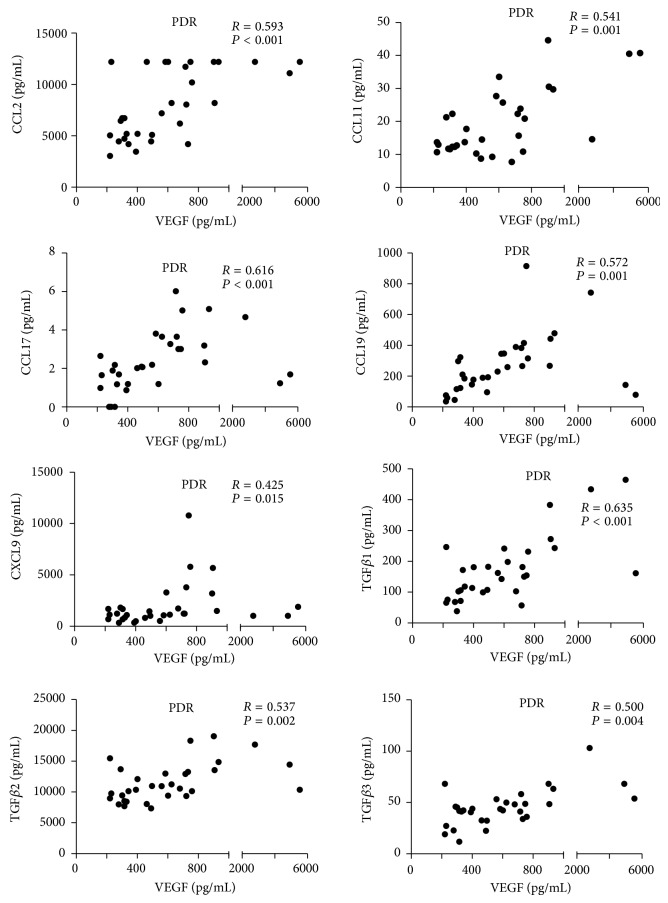
Correction analysis between VEGF and other mediators in PDR vitreous. Based on Spearman's analysis, moderate-to-strong corrections were observed between VEGF and CCL2, CCL11, CCL17, CCL19, CXCL9, TGF*β*1, TGF*β*2, and TGF*β*3.

In PDR eyes, strongly positive correlations were noted between VEGF and CCL17 (*r* = 0.616, *P* < 0.001) and between VEGF and TGF*β*1 (*r* = 0.635, *P* < 0.001). In PDR vitreous, moderate but significant associations were found between VEGF and CCL2 (*r* = 0.593, *P* < 0.001), CCL11 (*r* = 0.541, *P* = 0.001), CCL19 (*r* = 0.572, *P* = 0.001), CXCL9 (*r* = 0.425, *P* = 0.015), TGF*β*2 (*r* = 0.537, *P* = 0.002), and TGF*β*3 (*r* = 0.500, *P* = 0.004). The correlations of vitreous obtained from PDR patients were exhibited in Figure 2.

## 4. Discussion

In the present study, we investigated specific chemokines and growth factors in the vitreous humor of 58 eyes for associations with PDR. Using a multiplex bead immunoassay, we found that concentrations of all 11 chemokines and growth factors tested were significantly higher in the patients with PDR, relative to those without PDR. Our results showing the upregulation in PDR of CCL2, CXCL9, CXCL10, VEGF, TGF*β*1, and TGF*β*2 were in accordance with those of previous studies [2, 3, 15, 18, 19]. In addition, herein we were the first to report elevated levels of CCL17, CCL19, and TGF*β*3 in PDR eyes. We also found that CCL4 and CCL11 levels were significantly higher in PDR patients compared with the controls, which contradicts the reports of some others [2, 15, 17]. Moreover, CCL2, CCL11, CCL19, CXCL9, TGF*β*2, and TGF*β*3 levels moderately and positively correlated with those of VEGF, with stronger correlations between VEGF and CCL17, and between VEGF and TGF*β*1.

Currently, VEGF, known as a proangiogenic cytokine, has been recognized as the leading factor responsible for retinal neovascularization [2]. Elevated levels of VEGF in PDR vitreous were noted and short-term benefits of anti-VEGF agents for most PDR patients were observed [2, 9, 10]. However, about three in ten PDR patients exhibited insensitive to the initial anti-VEGF agents, indicating the requirement for rejected injection [2]. Additional concerns about ocular adverse effects and systemic adverse effects, such as hypertension, endophthalmitis, and retinal detachment, have been reported [11–13]. Thus, it is necessary to develop alternative therapeutic strategies, and we propose that the overexpression of chemokines and growth factors in PDR vitreous might be associated with retinal neovascularization. In the present study, via multiplex bead immunoassay, chemokines and growth factors related to PDR were identified.

Apart from attraction of leukocytes and amplification of local inflammation, chemokines played a critical role in the modulation of cell proliferation and retina neovascularization [1, 16, 20, 21]. In the present study, however, the origin and function of increased concentrations of chemokines remains to be clarified. The local resident cells and protein leakage were probably the two major sources of those raised chemokines. Here, we intended to focus on CCL4, CCL11, CCL17, and CCL19 in detail.

Yoshimura et al. [17] found that CCL4 and CCL11 were not present at detectable levels in PDR vitreous, while Bromberg-White et al. [2] reported that CCL4 and CCL11 were at low but markedly raised levels. In our study, however, the median CCL4 and CCL11 levels in PDR cases were approximately 1.3- and 1.2-fold higher, respectively, than the non-PDR cases.

More recently, Baier et al. [22] investigated the alteration of chemokines in colorectal carcinomas with respect to normal tissue. They found that CCL4 levels were obvious upregulated in colorectal carcinomas tissue as compared with controls [22]. Similarly, overexpression of CCL4 was observed in the tissues of renal cell carcinoma [23]. Surprisingly, in that study in patients at stages 1–3 with elevated CCL4 levels, the malignant tumor did not reoccur after radical surgery [23]. Thus, the investigators concluded that the antineoplastic effect of CCL4 was due to the suppression of angiogenesis in renal cell carcinoma [23]. More recently, Ishikawa et al. [24] have revealed that CCL4/CCR5 could trigger the angiogenic activity in hypoxic avascular retinas. They also pointed out that the modulation of CCL4 on pathological neovascularization depended on VEGF-A [24].

Recently, Georgiou et al. [25] indicated that angiogenesis-specific genes, such as CCL11, were markedly increased in plasma from patients with breast cancer. In human CCR3^+^ endothelial cells, CCL11 could directly activate and trigger the angiogenesis [16]. Also, the investigators demonstrated that it was through the induction of rat aortic sprouting that CCL11 exerted the angiogenic effect [16]. In addition, in a mice model of corneal angiogenesis, a significant elevation of CCL11 together with its receptor CCR3 was observed [26]. From another report of a mouse model of choroidal neovascularization administered a CCR3 antagonist, VEGF164 levels were markedly reduced in retinal pigment epithelial cells or choroid although no statistical difference in total VEGF expression was found [27]. Moreover, Wang et al. [14] showed that CCL11 could activate the VEGF receptor 2 (VEGFR2) signal via binding the specific receptor CCR3. The findings of the above studies, and ours in the present study that CCL4 and CCL11 levels were significantly higher in PDR patients than in the non-PDR controls, support our conclusion that CCL4 and CCL11 have an angiogenic effect on retinal neovascular diseases such as PDR.

In our study, CCL17 levels were statistically higher in PDR cases than controls. Weihrauch et al. [28] have shown that serum CCL17 levels were obviously elevated in the majorities of primary Hodgkin's disease. When patients completed the primary treatment, the median levels of CCL17 markedly dropped from baseline 5,803 pg/mL to 663 pg/mL [28]. In addition, compared with those with continuous complete response (CCR), CCL17 levels were statistically increased in individuals with progressive disease [28]. Thus, they hypothesized that CCL17 could act as the biomarkers for Hodgkin's disease [28]. Since the growth and metastasis of malignant tumor should be attributed to abnormal angiogenesis [29] and elevated expression of CCL17 was found in Hodgkin's disease [28], we speculated that CCL17 was essential for regulation of angiogenesis.

Usually, it is by binding specific receptor CCR7 that CCL19 exerts its biological effects [30, 31]. Of note, CCL19-CCR7 was reported to participate in the regulation of angiogenesis [32]. Pickens et al. [33] also indicated that CCL19 was associated with the angiogenesis in rheumatoid arthritis (RA). They found that in rheumatoid arthritis patients, CCL19 levels were significantly upregulated in synovial tissues compared with normal tissues [33]. Furthermore, formation of angiogenesis and pannus is a typical characteristic of RA. Pickens et al. [33] also believed that activation of CCL19 stimulated the production of VEGF. Supporting their findings, Brühl et al. [34] have shown that in RA or osteoarthritis (OA) patients, the role of fibroblast-like synoviocytes in the secretion of VEGF and the promotion of angiogenesis should be attributed to the stimulation of CCL19. Thus, CCL19 might be involved in PDR through the regulation of secretions of VEGF.

In the present study, TGF*β*3 levels were statistically different, with median levels of 43.02 pg/mL in PDR patients and 37.49 pg/mL in controls. In general, TGF*β*3 is principally derived from cells of mesenchymal origin [35]. Previous studies have shown that TGF*β*3 was observed at high levels in ocular anterior segment [36]. Also, TGF*β*3 was found mostly in isolated individual cells in the choroid and retina, such as microglia [37]. However, Tanihara et al. [38] have pointed out that gene expression of TGF*β*3 could not be confirmed in cultured retinal pigment epithelial cells (RPE). These observations have suggested that TGF*β*3 was most likely secreted by resident local cells of mesenchymal origin. Furthermore, protein leakage might be one source, due to the breakdown of blood-retinal barrier (BRB). Via the enhancement of angiogenesis and suppression of immune, upregulated expression of TGF*β* isoforms would promote the tumor cells survival [35]. Among three TGF*β* isoforms, TGF*β*3 were considered the most effective angiogenesis peptide [35]. Li et al. [35] further demonstrated that TGF*β*3 levels in plasma were significantly elevated in breast cancer patients with node metastasis, compared with those without node metastasis. Similarly and more recently, TGF*β*3 levels were obvious increased in plasma from patients with colorectal carcinoma, and TGF*β*3 levels were significantly higher in preoperative than in postoperative plasma samples [39]. Therefore, they proposed that TGF*β*3 could act as a potential marker of angiogenesis, especially for colorectal carcinoma patients [39]. In our current study, considering the relatively elevated levels of TGF-*β*3 in PDR samples, we concluded that TGF-*β*3 was linked to PDR, which is characterized by retinal neovascularization.

In the present study, moderate-to-strong correlations were noted between VEGF and others, which suggested a complex network of regulators in PDR. Of special note, CCL19 correlated positively with VEGF (*P* = 0.001, *r* = 0.572), and evidence has shown that CCL19 can stimulate the secretion of VEGF [34].

Our investigation indicated that a complex mix of chemokines and growth factors was involved in the process of retinal neovascularization. However, we only tested a small number of the chemokines and growth factors that were present in PDR vitreous, and there may be many others associated with the regulation of angiogenesis. For example, in a previous report, significant differences in interleukin (IL) 6 and IL8 levels between PDR and non-PDR patients were observed [2]. Similarly, Zhou et al. [40] demonstrated that inflammatory cytokines IL1*β*, IL6, IL8, and tumor necrosis factor (TNF) were markedly elevated in PDR vitreous, compared with controls. Recently, Semeraro et al. [41] reported that levels of erythropoietin (EPO) were statically higher in aqueous humor and vitreous of PDR patients than in patients with macular holes or puckers. Also, a significant correction was established between vitreous EPO and blood glucose in patients with PDR [41]. Moreover, adiponectin (APN) levels were markedly increased in aqueous humor obtained from PDR patients, and significantly decreased after injection of bevacizumab [42]. All these mediators may be potential targets in PDR treatment. More importantly, many additional cytokines, chemokines, and growth factors have not been measured, the mechanism of mediators related to PDR has not been completely elucidated, and it has not been determined which mediators independent of VEGF are the key factors responsible for the pathogenesis of PDR. These important questions leave much room for investigations.

Taken together, our primary data have shown that CCL2, CXCL9, CXCL10, VEGF, TGF*β*1, and TGF*β*2 levels were significantly increased in PDR vitreous compared with that of non-PDR, consistent with previous studies [2, 3, 15, 18, 19]. In addition, we report here first the overexpression of CCL17, CCL19, and TGF*β*3 in PDR eyes. CCL4 and CCL11 were also observed to be markedly upregulated in PDR vitreous. Most importantly, our results suggest candidates for further investigations of targeted therapy in PDR and the development of treatments that will avoid the adverse effects of anti-VEGF agents.

## Figures and Tables

**Table 1:** Clinical characteristics of the study population.

Clinical characteristics	Non-PDR *n* = 26		PDR *n* = 32	*P* value Non-PDR versus PDR
	MH *n* = 16	ERM *n* = 10		
Age (Y)				
Median (range)	59 (47–70)	56 (41–69)	58 (46–73)	*P* = 0.851
Gender				
Female	8 (50%)	6 (60%)	14 (44%)	*P* = 0.598
Male	8 (50%)	4 (40%)	18 (56%)	
Duration of Diabetic (Y)				
Median (range)	—	—	10 (5–14)	—
Fasting blood-glucose (mmol/L)				
Median (range)	4.84 (4.08–6.00)	5.09 (4.56–6)	7.35 (4.34–8.92)	*P* < 0.001
Glycosylated hemoglobin (%)	5.35 (4.78–5.8)	5.05 (4.67–6.0)	7.0 (4.7–9.6)	*P* < 0.001
Received insulin treatment	—	—	29 (90.6%)	—
Received photocoagulation treatment	—	—	28 (87.5%)	—

*P* value was calculated by Mann-Whitney *U* test or chi-square test between non-PDR and PDR cases.

**(a):** 

Chemokines	Detection ranges (pg/mL)
CCL2/MCP-1	1.92 pg/mL
CCL4/MIP-1*β*	1.59 pg/mL
CCL11	2.17 pg/mL
CCL17	0.66 pg/mL
CCL19	3.0 pg/mL
CXCL9	46.08 pg/mL
CXCL10	2.2 pg/mL

**(b):** 

Growth factors	Detection ranges (pg/mL)
VEGF	54.74 pg/mL
TGF*β*1	10.81 pg/mL
TGF*β*2	4.61 pg/mL
TGF*β*3	2.64 pg/mL

CCL: chemokine (C-C motif) ligand, CXCL: chemokine (C-X-C motif) ligand, VEGF: vascular endothelial growth factors, and TGF: transforming growth factor.

**(a):** 

Chemokines	Non-PDR *n* = 26	PDR *n* = 32	Mann-Whitney *U* test
CCL2/MCP-1 median (range)	1537 (902.07–3341)	7619.5 (3048–12191)	*P* < 0.001
CCL4/MIP-1*β* median (range)	17.86 (8.48–28.23)	23.22 (12.02–72.28)	*P* = 0.002
CCL11 median (range)	12.15 (4.62–18.84)	15.15 (7.72–44.61)	*P* = 0.003
CCL17 median (range)	ND	2.14 (0–6.02)	*P* < 0.001
CCL19 median (range)	41 (0–115)	219.67 (35.10–915.66)	*P* < 0.001
CXCL9 median (range)	200.44 (75.1–898.24)	1230.99 (340.37–10790)	*P* < 0.001
CXCL10 median (range)	284.82 (188.84–804.72)	1283 (327.13–15132)	*P* < 0.001

**(b):** 

Growth factors	Non-PDR *n* = 26	PDR *n* = 32	Mann-Whitney *U* test
VEGF median (range)	184.07 (51.54–273.39)	572.12 (221.54–5533)	*P* < 0.001
TGF*β*1 median (range)	16.71 (0–38.27)	157.98 (38.27–464.63)	*P* < 0.001
TGF*β*2 median (range)	7384.5 (4692–12261)	10743.5 (7347–19061)	*P* < 0.001
TGF*β*3 median (range)	37.49 (25.19–45.28)	43.02 (11.81–103.03)	*P* = 0.006

ND: not detected.
